# Tubal Ectopic Pregnancy: From Diagnosis to Treatment

**DOI:** 10.3390/biomedicines13061465

**Published:** 2025-06-13

**Authors:** Dimitrios Papageorgiou, Ioakeim Sapantzoglou, Ioannis Prokopakis, Eleftherios Zachariou

**Affiliations:** 1 Department of Gynecology, Athens Naval and Veterans Hospital, 115 21 Athens, Greece; 2 1st Department of Obstetrics and Gynecology, National and Kapodistrian University of Athens, 106 79 Athens, Greece; 3 1st Gynecology Department, Metropolitan General Hospital, 142 32 Athens, Greece

**Keywords:** ectopic pregnancy, tubal pregnancy, methotrexate, diagnostic laparoscopy, salpingectomy, salpingostomy, β-hCG, expectant management

## Abstract

The most frequent form of ectopic pregnancy, known as tubal pregnancy, leads to a dangerous situation where the fertilized ovum implants inside a fallopian tube, which can result in tubal rupture and severe bleeding. The purpose of this narrative review is to evaluate all existing data regarding epidemiology, risk factors, pathophysiology, clinical presentation, diagnosis, and management of tubal ectopic pregnancy in order to provide a comprehensive understanding of this common yet difficult clinical condition. Prior ectopic pregnancy, together with tubal pathology and assisted reproduction, represent the main risk factors for this condition. The diagnosis relies on serial β-hCG tests combined with transvaginal ultrasonography, but laparoscopy serves as the diagnostic tool for cases with uncertain results. The treatment plan depends on the fallopian tube integrity, along with the patient’s hemodynamic condition. Patients with unruptured pregnancies who are hemodynamically stable receive methotrexate treatment as the preferred option, but surgical intervention with salpingectomy or salpingostomy becomes necessary in case of tubal rupture or when medical treatment fails. The development of laparoscopic procedures has led to better results and improved possibilities for fertility preservation. The psychological effects on patients require both counseling and follow-up care. Early detection, along with personalized management, helps decrease maternal complications and optimize reproductive outcomes.

## 1. Introduction

Ectopic pregnancy (EP) is a significant medical entity during the reproductive period, characterized by the implantation of the embryo outside the endometrial cavity, most commonly in the fallopian tube. This condition remains one of the main causes of maternal morbidity and mortality worldwide. Although modern diagnostic and therapeutic modules have significantly improved the outcomes of affected cases, EP still poses a clinical and public health challenge, requiring careful analysis of risk factors, diagnostic options, and therapeutic approaches.

EP occurs in approximately 1–2% of all pregnancies, with recent data indicating an increase in its frequency due to the wider use of Assisted Reproductive Technologies (ART) and the increase in childbearing age [1]. According to the Centers for Disease Control and Prevention (CDC), the mortality associated with EP has significantly decreased in recent decades, primarily due to early diagnosis and prompt medical intervention [2].

The main types of EP include tubal, which accounts for approximately 95% of cases, as well as less common forms such as ovarian and abdominal pregnancy [3]. These forms vary in terms of frequency, pathophysiology, and therapeutic options, making an individualized strategy crucial for their management.

The clinical picture of an EP may include abdominal pain, abnormal vaginal bleeding, and feelings of dizziness or fainting. In severe cases, such as a ruptured fallopian tube, the symptoms may progress to hypotension and hemodynamic instability, making rapid diagnosis and immediate intervention critical to avoid life-threatening complications [1,4].

The diagnosis is based on the use of a combination of methods, such as the measurement of beta human chorionic gonadotropin (β-hCG) levels and transvaginal ultrasound. Continuous monitoring of β-hCG levels can help identify non-viable pregnancies, while ultrasound allows for the visualization of ectopic implantation [1].

Therapeutic options include medical, surgical, or conservative management, depending on the severity of the condition and the patient’s preferences. Methotrexate (MTX) medical therapy is the preferred method for stable patients with low β-hCG levels, while surgical interventions, such as salpingectomy or salpingostomy, are indicated in cases of rupture or hemodynamic instability [1,4].

Despite advances in diagnosis and treatment, EP still has long-term effects on reproductive health. Women with a history of EP are at increased risk of infertility and recurrences. Additionally, the experience of an EP can cause intense psychological distress, with increased levels of anxiety and worry about future pregnancies [5].

Research into the mechanisms leading to EP continues, revealing complex interactions between genetic, environmental, and hormonal factors. Understanding these processes facilitates the advancement of targeted preventive strategies and improved therapeutic interventions. For example, advancements in ART and improvements in surgical techniques can further reduce the risk of recurrence and improve reproductive outcomes [1].

The primary goal of the present narrative review is to collectively assess all the currently available data in terms of epidemiology, risk factors, pathophysiology, clinical appearance, diagnosis, and management options of tubal EP in an effort to provide a thorough understanding of such a not uncommon and still challenging clinical entity.

## 2. Definition, Epidemiology, and Risk Factors

A fertilized egg, after traveling through the fallopian tubes, normally attaches to the endometrial epithelium, which is located within the uterine cavity. The implantation of the blastocyst outside the uterine cavity is considered an EP.

About 95% of ectopic pregnancies occur in the fallopian tubes, making tubal implantation the most common form of ectopic pregnancy. The majority of tubal implantations occur in the ampulla region (70%), followed by isthmic pregnancy (12%), interstitial pregnancy (11%), and cornual pregnancy (2–3%). Less common sites of EP include the ovaries (3%), cervix (<1%), myometrium (<1%), caesarean section scar (<1%), and peritoneal cavity (<1%). EP occurs simultaneously with intrauterine pregnancy and is referred to as heterotopic pregnancy, with an incidence of 1 in 30,000 pregnancies.

The rates of EP and its complications vary greatly between various parts of Europe, Africa, Asia, and America. Demographic trends and disparities help explain how access to healthcare, social status, and cultural factors affect the rates of occurrence and treatment of ectopic pregnancies worldwide. It is estimated that EP accounts for 1–2% of all pregnancies globally [6], while in the US, it accounts for 0.5–1.5% of all first-trimester pregnancies [7]. This small fraction is responsible for 3% of all pregnancy-related mortality and 75% of first-trimester maternal mortality [8].

The incidence of EP has been associated with several risk factors. These risk factors affect the functioning of the fallopian tubes. Normally, a sperm fertilizes an egg in the ampulla of the fallopian tube and then travels through the tube to the implantation site, as explained above. Any process that interferes with the normal functioning of the fallopian tube during this process increases the chances of ectopic pregnancy. The underlying mechanism may be anatomical (e.g., scarring that hampers egg movement) or functional (e.g., decreased motility of the fallopian tube).

Gaskins et al., in their systematic review, identified a history of pelvic inflammatory disease, previous ectopic pregnancies, and certain surgical procedures in the female reproductive system as risk factors [9]. Repeated episodes of pelvic inflammatory disease due to Chlamydia trachomatis and Neisseria gonorrhoeae are well-documented causes of tubal damage and the formation of pelvic adhesions, which, in turn, cause EP [1,4]. A history of a previous EP and surgical interventions on the fallopian tubes for either fertility restoration or sterilization increase the risk of an EP. The likelihood of repeated EP increases up to eight-fold in comparison to those with no previous history of the condition. A study that involved over 1000 cases of EP demonstrated a recurrence rate of 10% [10].

In addition, age and smoking are related to a marked increase in the baseline risk [9]. The risk of EP is three times higher among women who smoke one pack of cigarettes per day or more. It is believed that the risk among smoking women who use ART is 15 times higher [1]. Sultana et al. also pointed out the role of socioeconomic status and healthcare availability, revealing that low-income groups have higher rates of ectopic pregnancies and often receive delayed care [11].

Demographic characteristics of the incidence of EP provide additional data on its epidemiology. Several studies conducted across different regions of the world show that age and ethnicity are two of the most critical factors. Higher maternal age has been associated with higher rates of ectopic pregnancies [12]. In India, the prevalence of ectopic pregnancies was found to be higher among women 30–35 years of age compared to younger women [12]. This association mostly has to do with age-related hormonal changes that alter the function and motility of the fallopian tube. In their study, Ganitha and Anuradha also established that demographic features, including place of residence, whether urban or rural, can influence the rate of EP. Lack of access to timely health care services in the rural areas further complicates the situation for the affected women, which points to the need for specific interventions [13]. ART has been recognized as a possible risk factor, and research suggests that the main cause of EP in women who have undergone IVF is of tubal origin. The incidence of EP following In Vitro Fertilization (IVF) is 1.7%. However, recent methods, such as transferring a 5-day blastocyst instead of a 3-day blastocyst, have been shown to lower the rates of the risk [14].

Contraceptive methods have been found to reduce the chances of conception and therefore the chances of ectopic pregnancy, but some forms of contraception may increase the risk of an EP if pregnancy occurs. The use of levonorgestrel-releasing intrauterine system (LNG-IUS) and a copper intrauterine device (IUD) increases the relative risk of EP [15,16]. According to the results of the study by Heinemann et al., 18% of the 61,448 women who used an IUD developed an EP [16]. This happens as a result of intrauterine devices that prevent intrauterine pregnancy, thus increasing the chances of implantation in an ectopic location. Similarly, progestin contraceptive tablets seem to have a small risk of increasing the rate of ectopic pregnancy because they affect the fallopian tubes by decreasing ciliary motility [16].

As already pointed out, anatomical changes in the fallopian tubes caused by peritubal adhesions as a result of appendicitis, salpingitis, or endometriosis, alongside congenital anatomical anomalies of the fallopian tubes mainly due to intrauterine exposure to diethylstilbestrol, are potential risk factors that may lead to EP. A list of risk factors is summarized in Table 1.

## 3. Pathophysiology

Hormonal regulation sets conditions for successful conception and supports the maintenance of pregnancy. The establishment of embryonic development, alongside endometrial modifications, becomes possible through human chorionic gonadotropin and progesterone during the first gestational stages. The inadequate hormonal release of human chorionic gonadotropin (hCG) and progesterone in the setting of an EP causes impaired trophoblast growth, along with adverse effects on tubal transport [15]. Furthermore, the altered hormonal environment might affect fallopian tube movement, which results in delayed ovum transfer to the uterus.

Normally, the junctional zone of the uterus produces regular contractions that help sperm move toward the ovum and guide the eutopic implantation of the embryo. Uterus contractility plays a pivotal role in the process of achieving pregnancy. According to a recent meta-analysis, clinical pregnancy rates of women undergoing assisted reproduction techniques decreased significantly when they displayed two or more uterine waves per minute during embryo transfer (OR 0.52, 95% CI 0.38–0.69) [17].

The contractility in women who suffer from medical conditions such as adenomyosis or those who have experienced ectopic pregnancies or infertility may become unpredictable by either speeding up or being uncoordinated. This dysregulated peristaltic activity results in functional stasis within the tubal–uterine junction, disrupts embryo transport, and increases the chances of ectopic implantation [18,19]. Transvaginal ultrasound and cine-MRI studies have shown irregular peristaltic myometrial wave patterns in women suffering from adenomyosis and endometriosis during the peri-ovulatory and luteal phases, which create barriers to embryo transportation during the critical window of implantation [20,21,22].

The observed uterine dysperistalsis functions as a crucial factor in tubal ectopic pregnancy pathogenesis since it may cause ectopic implantation [23]. Thus, the administration of spasmolytic agents alone or together with hormonal regulators has been proposed in order to reduce uterine wave frequency and improve implantation outcomes [23,24].

Similarly to the above, the fallopian tube transport irregularities play an essential role in the development of EP. The fallopian tubes include specialized structures that support the movement of gametes and the early embryo. Ectopic implantation develops when these mechanisms become disrupted. Tubal dysfunction commonly stems from pelvic inflammatory disease, as well as endometriosis and previous abdominal surgical procedures that create scarring and adhesions in the reproductive system [3]. Endometriosis, more specifically, creates an elevated risk of EP, as it damages the fallopian tube operation and produces an inflammatory response [1]. These anomalies become more complex when ART is involved because IVF elements increase the chances of ectopic implantation [1,2].

Impaired fallopian tube transfer can result in tubal rupture and tubal abortion, as well as the absorption of ectopic tissue. In cases of ruptured EC, the trophoblastic tissue invasion and the subsequent hemorrhage cause damage to the tube. The anatomy of the fallopian tube is an important factor in this process. The fallopian tube is devoid of a submucosal layer. The fertilized ovum easily passes through the epithelial layer to embed within the muscular wall of the fallopian tube. The trophoblast breaks down the underlying muscular tissue, and the rupture is spontaneous. The procedure may also be triggered by physical accidents, including bimanual vaginal examinations and sexual activities.

In cases of tubal abortion, the EC advances through the distal portion of the fallopian tube. The primary implantation site is crucial in this context. The hemorrhage may cease, and symptoms will gradually decrease, but this may continue if products of conception remain in the fallopian tube. Ectopic tissue absorption causes pregnancy to stop developing and then gets absorbed. The beta human chorionic gonadotropin levels show a declining pattern when concentrations are measured in this scenario.

The progression of an EP may evolve either acutely or chronically. The acute form of EP demonstrates elevated β-hCG concentrations at diagnosis, resulting in rapid diagnosis due to painful tube distension or underlying rupture. As such, tubal rupture probability remains higher in acute cases compared to chronic ones.

## 4. Clinical Signs and Symptoms

The clinical signs of an EP occur between the sixth and eighth weeks of gestational age. They can present later in the gestation, as discussed in the following chapters, in cases of ectopic pregnancies not located in the fallopian tubes. An EP is characterized by amenorrhea with some vaginal bleeding and pelvic or abdominal pain. It is also possible for the adnexal mass to be palpated. Symptoms such as nausea, breast tenderness, and breast engorgement are common in pregnancy and may also be present in cases of EP. The symptoms are usually not as severe as in other types of pregnancy, and this is attributed to the hormonal profile of EP, including the lower levels of β-hCG in women with EP compared to those with normal, uncomplicated pregnancy [3].

Vaginal bleeding is said to be lighter than the usual menstrual period and usually has a dark (brownish) discharge. It may start as spotting or brisk bleeding in severe cases. Hemorrhage or bleeding does not always imply EP, as it could be due to other conditions such as threatened abortion, viable early pregnancy, and trophoblastic disease. Bleeding with amenorrhea, especially in women with regular menstruation, makes the suspicion of EP more likely.

The pain may be either one-sided or two-sided and may range from mild to severe, worsening with physical activity or sexual intercourse. It is mostly located in the lower abdomen, and if it is diffuse, it may imply that the disease is life-threatening, requiring prompt action. In advanced cases where the fallopian tube ruptures, the symptoms may worsen to include abdominal pain, hypotension, and features of hypovolaemic shock. Pain may be radiated to the shoulder in cases of significant hemoperitoneum due to phrenic nerve irritation. These signs and symptoms are an indication for an immediate assessment due to the risk of severe morbidity and mortality [15,25].

## 5. Diagnostic Evaluation

### 5.1. Medical History and Clinical Examination

The primary symptoms of an EP consist of abdominal pain and irregular vaginal bleeding. However, patients may present with different levels of symptom severity.

The abdominal discomfort from EP tends to be unilateral, ranges from sharp to dull, and usually affects one specific area. Patients with this condition may experience widespread abdominal discomfort in addition to localized pain. The combination of symptoms creates diagnostic challenges because they match other conditions; thus, doctors might incorrectly identify the pain as non-gynecological. The severity of pain associated with an EP tends to change according to the condition’s progression, but it especially worsens when the sac ruptures, leading patients to seek emergency care [26].

Unusual vaginal bleeding stands out as the main symptom that helps doctors identify an EP. Vaginal bleeding from an EP shows different characteristics than regular menstrual blood and might be accompanied by dizziness or syncope if the patient is experiencing internal bleeding from a ruptured sac. Medical professionals need to evaluate the woman’s reproductive background while monitoring her hemorrhagic patterns since they may point to EP symptoms [27].

A detailed understanding of the patient’s history allows medical professionals to identify possible risk factors related to EP. Multiple demographic and clinical factors have proven essential in understanding the development of ectopic pregnancies. The combination of prior EP, along with pelvic inflammatory disease and underlying endometriosis, increases a woman’s chances of having an EP. Women who have undergone tubal ligation or tubal reanastomosis and other surgical procedures on their fallopian tubes become more prone to developing ectopic pregnancies [15].

### 5.2. Laboratory Evaluation

#### 5.2.1. β-hCG

The detection of EP by laboratory findings depends on serum β-hCG levels. β-hCG is a hormone that is produced during pregnancy and is used in the management of pregnancy complications. Modern pregnancy tests have very low detection thresholds. The detection level threshold in urine samples is between 20 to 25 mIU/mL, while in serum, detection is possible at levels less than 5 mIU/mL. The levels of β-hCG usually increase at a steady rate in normal intrauterine pregnancies, as it is usually observed that the levels double in early pregnancy over a period of about 48 h. In EP, the rise in β-hCG may be quite abnormal.

Some β-hCG levels can be useful in diagnosing EP. For instance, low levels of the hormone may indicate an abnormal pregnancy, while levels that have plateaued or show a decrease may signify that the pregnancy is not progressing as it should. High β-hCG values above the ultrasound discriminatory cutoff (approximately 1500–2000 mIU/mL) without visualization of an intrauterine pregnancy raise the suspicion of an EP. As such, the patterns of β-hCG levels may be used to distinguish between an EP and a normally progressing pregnancy [28].

The quantitative measurement of β-hCG in serum is important because it significantly contributes to the overall clinical evaluation. Thus, the concentrations of β-hCG, clinical features, and imaging studies are important for the diagnosis. For example, if a patient has abdominal pain, vaginal bleeding, and high β-hCG levels, the likelihood of an EP is high. However, it is important to note that β-hCG levels cannot be used to either confirm or exclude an EP on their own. Ultrasound is important in diagnosing EP, and β-hCG levels are used in conjunction with ultrasound findings to make a diagnosis.

The results of laboratory tests should be evaluated in the context of the whole clinical picture, as similar results may be seen in conditions such as miscarriage or trophoblastic disease. In cases of suspected EP, serum β-hCG levels are usually measured in serial samples to monitor hormonal trends that can help in assessing the viability of the pregnancy or the presence of ectopic gestation. The slow rise in β-hCG levels is concerning and warrants further action.

#### 5.2.2. Progesterone

Serum progesterone levels can be used in conjunction with the routine assessment of β-hCG and ultrasound to avoid the misdiagnosis of EP. If the ultrasound findings are indeterminate and the β-hCG levels are non-diagnostic, then an EP should be considered. In such cases, the serum progesterone levels can be used to support or exclude the diagnosis. A serum progesterone level of ≤6 ng/mL is consistent with a non-viable pregnancy, while a level of >25 ng/mL is consistent with a viable intrauterine one. It is important to note that progesterone levels alone cannot be used to diagnose ectopic pregnancy, and the values may not necessarily be indicative of ectopic as opposed to intrauterine pregnancy. Therefore, they should always be used in conjunction with clinical features, symptoms, and ultrasound findings [29,30,31].

### 5.3. Ultrasound Findings

The joint use of transabdominal and transvaginal ultrasound produces substantial information required to diagnose ectopic pregnancy. While the transabdominal ultrasound is an initial diagnostic modality, the transvaginal ultrasound improves diagnostic accuracy, particularly in early cases. Knowledge of imaging capabilities and restrictions helps physicians diagnose ectopic pregnancies effectively, a fact that leads to prompt medical interventions and improves patients’ outcomes. As such, the clinical practice employs both transabdominal and transvaginal ultrasound examinations.

In view of the above, transvaginal ultrasound stands as the superior diagnostic approach for identifying ectopic pregnancies at their initial stages. It shows superior ability in detecting small adnexal masses while providing clear differentiation between normal intrauterine pregnancies and EP. A detected anechoic or hypoechoic mass located near the tube or uterus enhances the probability of an ectopic implantation.

The diagnostic process requires ultrasound results, along with clinical signs and biochemical parameters, especially serum β-hCG, for complete evaluation. During an ultrasound examination, EP usually presents with the “cogwheel sign” as its distinctive feature. Such a sign is attributed to a gestational sac containing indistinct or partially developed fetal structures in the adnexa, while the presence of fluid in the Douglas pouch is indicative of a ruptured EP requiring emergency attention.

The suspicious ultrasound findings for EP include:

**Uterus**

1. Empty uterine cavity or absence of signs of intrauterine pregnancy: An exception to this is the rare case of heterotopic pregnancy.
2. Pseudogestational sac: Can be observed in 10–20% of ectopic pregnancies [32].
3. Pseudocyst: (Anechoic area that moves from the endometrial canal, mainly found at the junction of the endometrium and myometrium, indicating the formation of a decidual cast) [33].
4. Trifasic endometrium: (specificity 94%, sensitivity 38%) [33].

**Fallopian Tubes and Ovaries**

1. Simple adnexal cyst: 10% chance of EP.
2. Complex extrauterine cyst/mass: 95% probability of ectopic tubal pregnancy (if there is no intrauterine pregnancy).
  ➢ An intraovarian cyst/mass is more likely to be a corpus luteum.
3. Solid hyperechoic mass: Sensitive but nonspecific.
4. Ring sign (tubal ring sign/bagel sign/blob sign): An echogenic ring encircling an unruptured EP.
  ➢ 95% probability of a tubal EP if observed.
  ➢ It is described in 49% of ectopic pregnancies and in 68% of non-ruptured ectopic pregnancies.
5. “Ring of fire” sign [15]: Can be observed with color Doppler in a tubal EP, but also in a corpus luteum.
  ➢ The absence of flow on color Doppler does not exclude EP.
6. Positive cardiac function of an ectopic embryo: 100% specific but observed only in a small percentage of cases.

**Peritoneal Cavity**

1. Free fluid in the pelvis or hemoperitoneum in the Douglas pouch.
  ➢ Presence of free peritoneal fluid in combination with positive β-human chorionic gonadotropin (β-hCG) and an empty uterus (70% specificity, 63% sensitivity) [34].
  ➢ Nonspecific for ruptured EP (observed in 37% of non-ruptured tubal pregnancies).
2. Free fluid at the level of the hepatorenal recess.
  ➢ Free fluid in the Morrison’s pouch is a strong marker of the need for surgical intervention [35].
3. Cardiac activity in EP: 100% specific but observed only in a small percentage of cases.

In patients undergoing IVF, it is important to rule out the possibility of an EP, as the rate of heterotopic pregnancy in these patients is approximately 1–3:100, while the baseline risk of heterotopic pregnancy in spontaneous conceptions is minimal (1:30,000) [36].

### 5.4. Diagnostic Laparoscopy

Diagnostic laparoscopy stands as a vital diagnostic tool for both diagnosing and treating ectopic pregnancies because it offers several advantages over the traditional modalities of transvaginal ultrasound and serum β-hCG levels. The direct visual examination of pelvic organs through laparoscopy allows clinicians to verify EP with higher precision than non-invasive techniques. Yu et al. underlined that laparoscopic surgery provides not only an optimal diagnostic accuracy but also an evaluation of tubal patency, which matters for future reproductive planning. The procedure enables immediate surgical intervention, which has been associated with better outcomes [37].

Nonetheless, diagnostic laparoscopy possesses certain limitations. General anesthesia requirements create a fundamental challenge because specialist equipment and trained personnel availability remains restricted in certain regions, particularly in countries with limited resources. The surgical results obtained from laparoscopy heavily depend on both the surgeon’s level of expertise and their technical skills [38]. In view of the above, the implementation of diagnostic laparoscopy for EP diagnosis represents a significant advancement that has improved treatment standards and patient results despite its limitations [37,39].

### 5.5. Diagnostic Algorithm

The patient’s clinical symptoms and signs guide the diagnostic procedures when serum β-hCG amounts are below 1500 mIU/mL. Patients showing multiple signs and symptoms of EP and hemodynamic instability should undergo laparoscopy.

Patients who show few symptoms can receive serial β-hCG assessment as part of their conservative care. The β-HCG level in normal pregnancies should double every 48 h, and the suspicion of an atypical pregnancy develops when β-HCG levels fail to follow that pattern while the location of the pregnancy remains unknown [1].

When β-HCG levels fail to double every 48 h or even decrease, cervical dilatation and uterine curettage may be employed, which may reveal intrauterine gestational components (chorionic villi) through histological examination. The diagnosis of EP becomes likely when postprocedure β-hCG levels show less than 15% decline after 12 h or when chorionic villi are absent in histology examinations.

**Figure 1:**
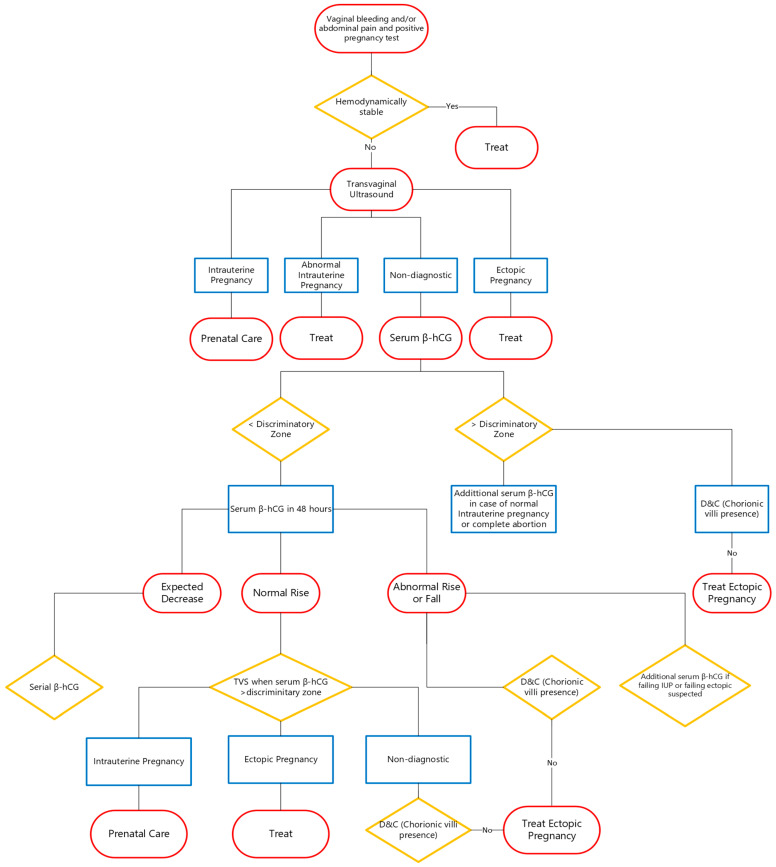
Diagnostic algorithm for suspected EP.

The diagnostic process for suspected EP is presented in detail in Figure 1 [40].

## 6. Treatment Modalities

The therapeutic approach might be based on three primary modalities. The options include surgical intervention, medical management with MTX, and expectant management. It is important to acquire the Rhesus status of the patient before initiating any of the above-mentioned therapeutic strategies in order to avoid the alloimmunization of Rh-negative patients. As such, it is advisable to administer 300 μg of anti-D immunoglobulin when the paternal Rh status is unknown or if the paternal Rhesus status is positive.

### 6.1. Medical Management

MTX has been recently established as the standard treatment for EP, especially in hemodynamically stable patients who are not at risk of tubal rupture. The medication works by inhibiting cell proliferation and inducing apoptosis in proliferating trophoblastic tissue, which makes it very useful for the management and treatment of early EP. In a meta-analysis comparing expectant management with MTX treatment in unruptured EP, the success rates for MTX treatment were approximately 90%, whereas those of expectant management were approximately 70%. Furthermore, MTX treatment was related to lower recurrence rates [41].

Research is currently ongoing in order to explore new drug regimens and other medications that may help improve pharmacological treatment. A new pharmacological regimen includes the administration of mifepristone and MTX for non-tubal ectopic pregnancies. A pilot study was conducted to evaluate the effectiveness and safety of the mifepristone and MTX combination, based on the rationale that mifepristone may enhance the efficacy of MTX by inhibiting further trophoblast growth, with the results revealing that the combination therapy had higher success rates than MTX monotherapy [42].

Although surgical management appears to be the most suitable option, the option of MTX treatment may be able to prevent the need for surgery. However, there are certain conditions that must be met for its administration. Patients should be hemodynamically stable, and there should be no evidence of predisposition to tubal rupture. Furthermore, there must be no contraindications to the administration of MTX (Table 2), while patients should be educated on the potential side effects and complications of the drug, such as stomatitis, liver failure, bone marrow suppression, pneumonitis, and allergic reactions [4].

In addition, patients who choose MTX treatment for EP must have timely access to an emergency department for the early diagnosis and management of potential complications and must adhere strictly to the recommended follow-up, which includes blood samples for repeated β-hCG measurements [4].

Furthermore, MTX is advised as a therapeutic option for women with particular characteristics conducive to the resolution of EP, as outlined in Table 3. Upon the fulfillment of these parameters, MTX treatment demonstrates efficacy in around 95% of instances.

MTX for the treatment of EP exists in four forms, which include intravenous and intramuscular administration, as well as oral administration and transvaginal or transabdominal gestational sac injection. The predominant method of administration is intramuscular. The success of MTX injection into the gestational sac depends heavily on the practitioner’s skills. Research findings showed that intrasac MTX administration might work better than systemic administration, but the results failed to reach statistical significance [43].

Three different MTX treatment protocols exist in international medical literature, which include one-dose, two-dose, and multiple-dose regimens [1].

The single-dose approach stands as the most commonly used, while also representing the simplest method for implementation. However, it may require an additional repeat dose to ensure complete resolution of the EP. The two-dose protocol follows the same follow-up β-hCG level monitoring as the single-dose protocol but includes a second MTX dose on day four. The multiple-dose protocol requires folic acid administration with MTX to reduce its adverse effects. The treatment duration of this regimen can reach up to 8 days [1,4,15]. The characteristics of the above-mentioned protocols are summarized in Table 4.

Patients need to know that fertility and ovarian reserve are unaffected by MTX therapy [4]. An EP has a recurrence rate of about 15% after a single prior episode of EP, and it rises up to 30% after two previous cases of EP. The risk of recurrence appears to be the same whether the intervention is medical or surgical. High levels of β-hCG (above 5000 mIU/mL) may increase the risk of a subsequent tubal occlusion, regardless of the drug.

It is possible to have another EP after the first one has occurred. The risk of another EP is about 15 percent, and this risk increases to 30 percent after two ectopic pregnancies. The risk of recurrence appears to be the same for medical and surgical interventions [1]. β-hCG levels higher than 5000 mIU/mL may be associated with an increased risk of subsequent tubal occlusion, regardless of the treatment modality used [44].

### 6.2. Surgical Management

The decrease in importance of surgical treatment of EP is related to the development of pharmacological treatments when ectopic pregnancy has not ruptured. Nonetheless, there are some women who opt for surgery or are unsuitable for pharmacological treatment. For example, women with serum β-hCG values above 5000 IU/L or those in whom fetal cardiac activity is detected on ultrasound are more likely to be operated on, given the anticipated high failure rate of MTX treatment. In this context, surgical intervention should be undertaken only in hemodynamically stable patients with a clearly diagnosed EP by transvaginal ultrasonography, while in inconclusive cases, medical treatment or expectant management with regular transvaginal ultrasounds should be preferred.

Nevertheless, there are some circumstances in which surgical treatment is preferable to pharmacological therapy, and if these criteria are fulfilled, surgical therapy should be considered the preferred one (Table 5).

For the treatment of EP, the following surgical techniques are recommended: salpingectomy, which is the total excision of the fallopian tube or a part of it, and salpingostomy, which is the excision of the EP while keeping the fallopian tube in situ. The patient’s general well-being, the patient’s desire to have children in the future, and the extent of fallopian tube damage are all important factors that will help decide on the best course of action for each patient.

Randomized clinical studies have shown that there is no significant difference in the rate of future intrauterine pregnancy between patients who had salpingectomy and those who had salpingostomy. On the other hand, prospective studies have shown that salpingostomy is associated with higher rates of recurrent EP [45].

Salpingostomy is recommended for hemodynamically stable patients with a desire for future pregnancy when the contralateral tube is affected, as well as in patients in whom the removal of the tube would necessitate IVF for subsequent conception. In salpingostomy with suspected retained pregnancy products in the tube, a prophylactic dose of MTX postop is recommended [46].

Laparoscopic intervention is the best treatment modality in cases of EP. The majority of EPs, including cases of heterotopic and interstitial pregnancies, as well as cases of hemoperitoneum, can be effectively managed by the laparoscopic technique. The choice of the appropriate surgical method depends on the skill of the surgeon and anesthesiologist, as well as the patient’s condition. In patients who show hemoperitoneum on ultrasonographic examination, bleeding occurs gradually. The surgical method (laparoscopic or open, salpingostomy or salpingectomy) in these cases is decided by the surgeon’s ability to perform laparoscopic surgery and the anesthesia team’s level of readiness and expertise.

In view of the above, a systematic review of randomized controlled trials comparing laparoscopic salpingostomy with open salpingostomy found that the laparoscopic approach resulted in less operating time (73 min vs. 88 min), less blood loss (79 mL versus 195 mL), and shorter hospital stay and recovery time (1–2 days versus 3–5 days and 11 days versus 24 days, respectively). The study found that the laparoscopic method had higher rates of residual disease than the open procedure; however, the rates of recurrent EP were not significantly different. The high incidence of retained products of conception is mostly due to the surgeon’s (in)experience and the surgical procedure itself, as the partial removal of products of pregnancy with forceps increases the likelihood of residual disease. Fluid irrigation under pressure is the recommended technique for their removal [43].

The efficiency of laparoendoscopic single-site surgery (LESS) in relation to traditional laparoscopic salpingectomy seems to offer no advantage in terms of surgical time, hemorrhage, or length of hospital stay [47]. Similar to medical treatment follow-up, β-hCG levels should be monitored after surgery. Weekly β-hCG measurements are recommended after laparoscopic salpingostomy to rule out residual disease. The decrease in β-hCG after salpingectomy is similar to that observed after salpingotomy. A review of 147 EP cases treated surgically revealed that a decrease in β-hCG by more than 76% on the first day postoperatively was consistent with no remaining ectopic tissue. The same study revealed that on the first postoperative day, β-hCG decreased by more than 50% compared to the preoperative levels. [48] Alternatively, as mentioned above, should there be suspicion of retained products of conception, the administration of a prophylactic dose of MTX postop is recommended [46].

There is insufficient evidence in the literature to suggest the optimal period between surgery and the attempt at subsequent pregnancy. It is recommended that attempts to conceive may be initiated after the first menstruation following the surgery. Randomized controlled trials showed that there was no significant difference in the patency of the fallopian tubes, the incidence of recurrent ectopic pregnancy, or future fertility between MTX treatment and laparoscopic intervention with tubal preservation. [4,43]. Pharmacological treatment is considered cost-effective when β-hCG levels are less than 1500 mIU/mL and when laparoscopy is not required for diagnosis. However, when the duration of the pharmacological treatment for the complete resolution of the EP is anticipated to be long or when there is a high likelihood of medical treatment failure, surgical intervention is preferred. This includes cases in which the ectopic embryo has cardiac activity or the β-hCG is elevated or rising [1,4].

### 6.3. Expectant Management

Expectant management in cases of EP can only be considered in asymptomatic patients, with evidence of resolving EP and being fully aware of the impending risks of tubal rupture, hemorrhage, and, possibly, the need for emergency surgical intervention. Studies have revealed that 88% of EPs with initial β-hCG levels less than 200 mIU/mL will spontaneously resolve [1,4].

It is important to closely observe those patients, as there have been cases of tubal rupture reported in cases with low β-hCG levels. It is prudent that the β-hCG should be checked at least three times, 48 h apart from each other, to ensure that the levels are declining [1,4]. It is recommended that β-hCG should be measured once a week until the levels become zero, when the risk of rupture is low. In asymptomatic women, expectant management may be continued if the β-hCG levels are falling or have reached stable levels, with the option of administering a dose of MTX.

A randomized trial showed that expectant management as a treatment modality was not any less effective in a statistically significant way than MTX treatment for managing EP in women with β-hCG less than 2000 mIU/mL, as its success rate was 59% compared to 76% when MTX was used [49]. Due to the difficulties in identifying the right cohort of patients and the issues of safety and effectiveness, such an approach is not often used in the management of EP. The rates of spontaneous resolution of ectopic pregnancies under expectant management are reported to be between 30% and 70%, rates that range depending on the selection criteria [50,51]. However, this approach should be discontinued if the woman has persistent discomfort, fails to show a decline in β-hCG levels in successive measurements, or there are clinical findings suggestive of tubal rupture and/or hemoperitoneum [4].

β-hCG, particularly its initial levels, may be used as a predictor of the likelihood of spontaneous resolution of an EP [51]. The probability of spontaneous resolution of EP is as high as 96% for women with initial β-hCG levels below 175 IU/L; on the other hand, treatment failure is observed in 93.3% of those with initial β-hCG levels above 2000 IU/L. Furthermore, among patients with initial β-hCG levels between 175 and 1500 IU/L, a serum progesterone concentration less than 10 nmol/L, and a gestational age of less than 42 days, spontaneous resolution was observed in 66% of cases [50,51].

Recent research demonstrates that initial β-hCG measurements determine which patients with ectopic pregnancies can safely undergo expectant management. A 2023 retrospective cohort study demonstrated that patients who had discharge β-hCG levels under 650 IU/L and showed at least a 50% decrease in β-hCG levels during hospitalization achieved a 97% success rate with expectant management. The success rate for patients with discharge β-hCG levels at 1000 IU/L or higher remained at 50% regardless of their β-hCG decline rate [52].

Another study utilizing a machine learning approach reported that patients with β-hCG levels under 1000 mIU/mL achieved an 80% success rate regardless of their β-hCG decline rate. The success rate for patients with β-hCG levels above 1000 mIU/mL remained equivalent when their β-hCG levels decreased by more than 15%. The research indicates that patients with low initial β-hCG values who experience substantial early reductions in their β-hCG levels tend to succeed with expectant management. Expectant management remains an option for patients with elevated initial β-hCG levels when there is substantial early decline but requires increased vigilance and close observation [53]. In the context of fertility preservation after expectant management, the hysterosalpingography revealed tubal patency in 93% of the cases. Research has shown that the success rate of achieving an intrauterine pregnancy using such an approach ranges from 63% to 88% [54].

## 7. Νovel Insights, Current Research, and Future Directions

Recent research has focused on the investigation of novel markers and their additive yield on the diagnosis of EP. Creatine phosphokinase (CPK) has been thoroughly assessed to evaluate the impact of its serum levels on the early diagnosis of EP, with studies producing contradicting results [55,56,57]. However, a recent systematic review that included all studies that explored the diagnostic yield of CPK in the early diagnosis of EP demonstrated its overall potential benefit [58]. Similar to CPK, several additional markers associated with the dysregulation of the trophoblast function (activin) [59] or blastocyst tubal transport (CB1) [60], along with a number of proteins (ADAM12, pregnancy-associated plasma protein A, fibronectin), have been investigated in recent years, but none of them has yet been validated or implemented in current clinical practice [61,62,63]. Only recently, Turkoglu et al. further deepened the research on EP by investigating a series of metabolites associated with the development of underlying inflammation and atypical placentation in an effort to assess their involvement in the detection of tubal EP [64].

Current research has also focused on novel potential treatment modalities in view of novel surgical and medical approaches. Xiao et al. have recently initiated a randomized controlled trial to assess the applicability and safety of Transvaginal Natural Orifice Transluminal Endoscopic Surgery for Tubal Ectopic Pregnancy (vNOTESTEP) as an alternate modality to the traditional laparoscopic approach [65]. In terms of medical management, letrozole has been investigated as an alternative drug regimen to methotrexate with a recent meta-analysis including a total of 152 patients, demonstrating that letrozole is more effective than surgery with equivalent efficacy to methotrexate [66]. However, the authors underlined the potential presence of bias in the included studies and that results should be viewed with caution, a fact that was further highlighted by the results of a recent prospective cohort study [67].

## 8. Conclusions

Ectopic pregnancy remains a potentially life-threatening condition in modern gynecology. Tubal ectopic pregnancy, as the most common type of EP, may jeopardize maternal health, resulting in increased rates of morbidity and mortality. This review is intended for obstetricians, gynecologists, emergency physicians, and fertility specialists who manage tubal ectopic pregnancies. Clinicians can reduce complications and enhance outcomes in tubal EP by using evidence-based diagnostics, tailored treatments, and multidisciplinary follow-up.

Specifically, a combination of clinical assessment, laboratory indices, and imaging modalities to achieve a timely diagnosis and determine the appropriate treatment strategy will enhance survival and minimize adverse effects. If left untreated, the likelihood of rupture increases with advancing gestational age, leading to a series of severe consequences and adversities. Expectant management can be quite beneficial in cases of suspected early abortion, whereas medical management is the most appropriate approach that should initially be used when the appropriate criteria are met. However, only surgical intervention can deal with emergency catastrophic events. Future research should concentrate on new biomarkers, less invasive surgical methods, and optimal fertility preservation approaches to enhance patient care.

## Figures and Tables

**Table 1:** Ectopic pregnancy risk factors [1,2,4,15,16].

History of ectopic pregnancy.
History of surgery on the fallopian tubes.
Smoking more than 20 cigarettes daily.
PID confirmed through laparoscopy or a positive test for Chlamydia trachomatis.
Three or more prior miscarriages.
Individuals aged 40 years or older.
History of medical or surgical abortion.
Infertility lasting more than one year.
More than 5 sexual partners.
Previous use of IUD.
Initiation of romantic relationships during adolescence.

Abbreviations: PID: pelvic inflammatory disease; IUD: intrauterine device.

**Table 2:** Contraindications in MTX administration [1,4,15].

Absolute Contraindications	Relative Contraindications
Intrauterine pregnancy	Fetal heart activity detectable on transvaginal ultrasound
Immunodeficiency	Increased hCG
Moderate to severe anemia, leukopenia, thrombocytopenia	EP larger than 4 cm
Sensitivity to MTX	Refusal to accept a blood transfusion
Active pulmonary disease	
Active peptic ulcer	
Liver failure	
Renal failure	
Ruptured EP	
Breastfeeding	
Hemodynamic instability	
Inability to participate in the follow-up	

Abbreviations: MTX: methotrexate; EP: ectopic pregnancy; hCG: human chorionic gonadotropin.

**Table 3:** Favorable conditions for administering treatment with ΜΤΧ [4,25].

β-hCG < 5.000 IU/L
Diameter of EP < 35 mm
Absent cardiac activity in the TVUS

Abbreviations: MTX: methotrexate; β-hCG: beta human chorionic gonadotropin; EP: ectopic pregnancy; TVUS: transvaginal ultrasound.

**Table 4:** Pharmacological protocols for EP management [1,4,15].

	Single Dosing Protocol	Multiple Dosing Protocol
**Doses**	1 dose. Administer an additional dose if necessary.	Up to 4 doses until the serum β-hCG levels decrease by 15%.
**Dosage**		
*Methotrexate*	50 mg/m^2^ Body Surface Area (Day 1).	1 mg/m^2^ on Days 1, 3, 5, and 7.
*Leucovorin*	Not administered.	0.1 mg/m^2^ on Days 2, 4, 6, and 8.
**Assessment of serum β-hCG concentrations**	Days 1, 4, and 7.	Days 1, 3, 5, and 7.
**Recommendation for a supplementary dosage**	1. If the β-hCG levels do not decrease by 15% between D4 and D7. 2. If the reduction in β-hCG levels is less than 15% between weekly assessments.	If the β-hCG levels decline by less than 15% between D1 and D3, an extra dose is given. A serum β-hCG level measurement is repeated and compared with the prior value after 48 h. Maximum dosage: 4 doses.
**Follow-up**	Once a 15% reduction in β-hCG levels is achieved, weekly evaluations of serum β-hCG levels will continue until they are undetectable.	

Abbreviations: EP: ectopic pregnancy; β-hCG: beta human chorionic gonadotropin; D: day.

**Table 5:** Indications for surgical intervention in EP [1,4,15,40].

Hemodynamic instability	Desire for permanent contraception
Impending or ongoing rupture of an EP	Known tubal factor in patients planning to undergo IVF
Concurrent presence of intrauterine pregnancy	Failure of medical therapy
Contraindications for the administration of MTX	
Failure to adhere to medical management and/or inability to have consistent monitoring post-treatment	

Abbreviations: EP: ectopic pregnancy; MTX: methotrexate; IVF: In Vitro Fertilization.

## Abbreviations

The following abbreviations are used in this manuscript:

EP	ectopic pregnancy
CDC	Centers for Disease Control and Prevention
LNG-IUS	levonorgestrel-releasing intrauterine system
IUD	intrauterine device
ART	Assisted Reproductive Technologies
IVF	In Vitro Fertilization
β-hCG	beta human chorionic gonadotropin
IUP	intrauterine pregnancy
LESS	laparoendoscopic single-site surgery
TVUS	transvaginal ultrasound
PID	pelvic inflammatory disease
CPK	creatine phosphokinase
